# Endothelial cell regulation of leukocyte infiltration in inflammatory tissues

**DOI:** 10.1155/S0962935195000524

**Published:** 1995-09

**Authors:** A. Duperray, A. Mantovani, M. Introna, E. Dejana

**Affiliations:** 1 CEA Laboratoire d'Hématologie INSERM U 2 17 DBMS CEN-G Grenoble France; 2 Istituto di Ricerche Farmacologiche ‘Mario Negri’ Milano Italy; 3 Section of General Pathology Dept of Biotechnology Università di Brescia Italy

## Abstract

Endothelial cells play an important, active role in the onset and regulation of inflammatory and immune reactions. Through the production of chemokines they attract leukocytes and activate their adhesive receptors. This leads to the anchorage of leukocytes to the adhesive molecules expressed on the endothelial surface. Leukocyte adhesion to endothelial cells is frequently followed by their extravasation. The mechanisms which regulate the passage of leukocytes through endothelial clefts remain to be clarified. Many indirect data suggest that leukocytes might transfer signals to endothelial cells both through the release of active agents and adhesion to the endothelial cell surface. Adhesive molecules (such as PECAM) on the endothelial cell surface might also ‘direct’ leukocytes through the intercellular junction by haptotaxis. The information available on the molecular structure and functional properties of endothelial chemokines, adhesive molecules or junction organization is still fragmentary. Further work is needed to clarify how they interplay in regulating leukocyte infiltration into tissues.


Mediators of Inflammation 4, 322–330 (1995)

### Endothelial cell regulation of leukocyte infiltration in inflammatory tissues

A. Duperray,[1,CA] A. Mantovani,[2,3] M. Introna[2] and E. Dejana[1]

[1]CEA, Laboratoire d'Hématologie, INSERM U 217 DBMS CEN-G, Grenoble, France; [2]Istituto di Ricerche Farmacologiche 'Mario Negri', Milano, Italy and [3]Section of General Pathology, Dept of Biotechnology, Università di Brescia, Italy

[CA]Corresponding Author

**Endothelial cells play an important, active role in the onset and regulation of inflammatory and immune reactions. Through the production of chemokines they attract leukocytes and activate their adhesive receptors. This leads to the anchorage of leukocytes to the adhesive molecules expressed on the endothelial surface. Leukocyte adhesion to endothelial cells is frequently followed by their extravasation. The mechanisms which regulate the passage of leukocytes through endothelial clefts remain to be clarified. Many indirect data suggest that leukocytes might transfer signals to endothelial cells both through the release of active agents and adhesion to the endothelial cell surface. Adhesive molecules (such as PECAM) on the endothelial cell surface might also 'direct' leukocytes through the intercellular junction by haptotaxis. The information available on the molecular structure and functional properties of endothelial chemokines, adhesive molecules or junction organization is still fragmentary. Further work is needed to clarify how they interplay in regulating leukocyte infiltration into tissues.**

**Key words:** Adhesion molecules, Chemokines, Cytokines, Diapedesis, Endothelium, Extravasation, Inflammation, Leukocytes.

### Introduction

Circulating leukocytes migrate from the vessels and enter tissues under normal or pathological conditions. Whereas monocytes, lymphocytes and natural killer cells exhibit a significant spontaneous migration through resting endothelial cells (EC), neutrophils and eosinophils require chemotactic stimuli and/or endothelial cell activation.[1–3] Cell migration across endothelial monolayers involves leukocyte adherence to the endothelium, crawling on the endothelial surface and penetration between endothelial clefts.

Endothelial cells can actively regulate leukocyte infiltration in inflammatory tissues through different mechanisms such as vasodilatation, release of chemotactic cytokines, expression of adhesion molecules and opening of interendothelial junctions (Fig. 1).[1,2,4–6]

All these reactions act in concert in localizing leukocytes and in facilitating their passage through the interendothelial junctions. Inflammatory stimuli are able to activate endothelial cells inducing their functional reprogramming toward a 'proinflammatory' phenotype.[5] Interleukin-1 (IL-1) or tumour necrosis factor (TNF) induce production of the vasodilatatory mediators such as prostacyclin and nitric oxide,[5,7,8] as well as the synthesis of a large series of adhesive molecules[1] and chemokines[5], and cause endothelial increase in permeability and alteration of junction organization.[6,9,10] In addition to 'classic' inflammatory agents, stimuli associated with the development of atherosclerotic plaques (such as minimally oxidized low-density lipoprotein [LDL]) can also modify endothelial cell reactivity and induce monocyte and lymphocyte infiltration into the vessel wall.[11] In a general sense atherosclerotic plaque evolution presents many similarities with inflammatory reactions.

In this review we will focus concisely on the role of endothelial cells in promoting leukocyte infiltration in tissues. In particular, we will consider chemokine production, expression of adhesive molecules and regulation of junction organization. Previous reviews of this rapidly expanding area of research provide the background and framework for this contribution.[1,6,10,12,13]

### Chemokines

The proinflammatory chemokines are a family of 16 homologous low molecular weight (8–10 kDa) proteins. They activate different leuko-

© 1995 Rapid Science Publishers

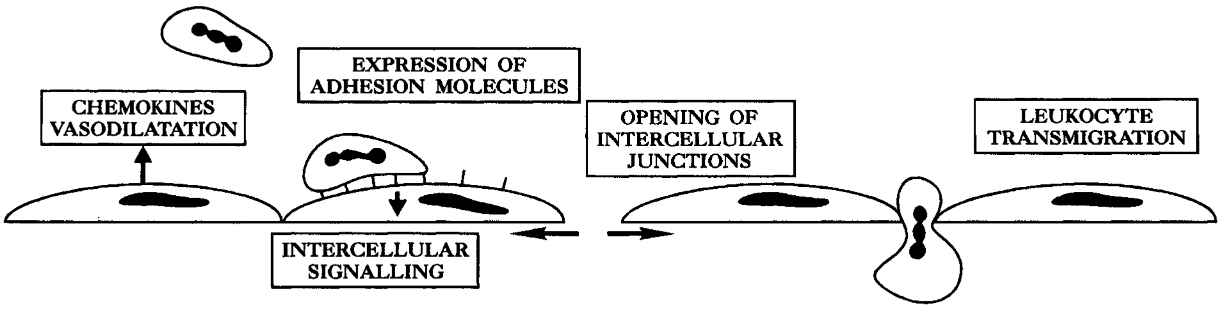

FIG. 1. Leukocyte extravasation as a multistep process regulated by the endothelium.

Table 1. The chemokine family

| α-chemokines (C–X–C) | β-chemokines (C–C) |
|---|---|
| IL-8 | RANTES |
| GRO-α-β,-γ | MCP-1,-2,-3 |
| IP-10 | MIP-1α |
| ENA-78 | MIP-1β |
| MGSA | |
| NAP-2 | |

For details see References 14 and 15.

cyte subtypes inducing a large set of responses including change in cell shape, release of enzymes, formation of bioactive lipids, respiratory burst and most importantly activation of adhesive molecules and chemotaxis.[14,15] One of their characteristics is the presence of four cysteines, conserved in all members of the family. They can be subgrouped in α-chemokines (Table 1) when the first two cysteines are interrupted by one amino acid (C–X–C) and β-chemokines when they are together (C–C). α-Chemokines act on neutrophils, β-chemokines activate monocytes, eosinophils and basophils.[14,15]

*In vitro studies:* Endothelial cells produce various chemokines in response to signals representative of inflammatory reactions, immunity and thrombosis.[5] Inflammatory cytokines (IL-1 and TNF) and bacterial endotoxins induce expression and release of IL-8 and GROα.[16–23] Induction of IL-8 expression is associated with and depends on gene transcription.[20] IL-4 and IL-13 are weak inducers of IL-8 expression and amplify induction by inflammatory cytokines.[24,25–28] Histamine induces IL-8 production in EC.[29] Hypoxia has recently been shown to induce IL-8 and MCP-1 expression in EC, a finding potentially relevant for pathological conditions in which activation and recruitment of leukocytes may amplify tissue damage.[30,31] Platelets contain IL-1 and, when they interact with vascular EC, induce IL-8 gene expression.[32]

As a result of proteolytic cleavage, IL-8 versions with a different $NH_2$ terminus and length can be produced.[14] It has been suggested that EC release predominantly a 77-amino acid version of IL-8, which is a less active species at activating leukocytes than the most common 73-residue form.[23,33] The proteolytic conversion to smaller versions of the molecule can be catalysed by thrombin.[33]

The influence of IL-8 on the interaction of polymorphonuclear cells with vascular EC has been the object of seemingly conflicting observations, which seem now to reflect different experimental protocols and, most interestingly, different functions exerted by this cytokine under different pathophysiological conditions. IL-8 increased the adhesiveness of normal polymorphonuclear leukocytes for normal EC.[34] In apparent contrast with these findings, EC-derived IL-8 was reported to inhibit binding of the polymorphonuclear leukocytes to activated EC.[23] Although it elicits polymorphonuclear leukocyte extravasation when given locally, IL-8 inhibits recruitment if administered systematically by the i.v. route.[35,36] The seemingly paradoxical anti-inflammatory effects of high levels of systemic IL-8, possibly dependent upon the action of a reverse chemotactic gradient and leukocyte deactivation, may represent a feedback mechanism to control tissue damage.

The role of IL-8 produced locally by vascular cells was reexamined using reconstructed vessel wall models.[37] Unequivocal evidence was obtained for the importance of IL-8 in transendothelial migration induced by inflammatory cytokines.[37,38]

EC of postcapillary venules bind IL-8, possibly via heparin-like molecules.[39] EC of postcapillary venules in kidney and other tissues express the promiscuous chemokine receptor present also on erythrocytes and known as Duffy antigen.[40,41] This receptor is present on EC of both Duffy+ and Duffy− individuals and may serve to present chemokines to circulating leukocytes. Solid phase IL-8 elicits haptotactic migration.[42] Thus, locally produced IL-8 may be retained on the surface of EC and activate adhesive interactions and migration.[39]

EC activated *in vitro* by inflammatory cytokines express GRO$\alpha$, which according to one report, could in turn act on EC.[17] It has been suggested that EC-bound GRO$\alpha$ may promote monocyte adhesion.[43]

IP10, a member of the C–X–C family but unique in that it attracts monocytes, is expressed in certain endothelia of mice exposed *in vivo* to IFN$\gamma$ or to lipopolysaccharides (LPS).[44,45] There are no reports on *in vitro* expression of this chemokine in EC.

EC produce substantial amounts of the C–C chemokine MCP-1.[21,22] The proinflammatory signals IL-1, TNF and, to a lesser extent, endotoxin are potent stimuli for MCP-1 production.[21,22] IL-4 and IL-13 are active, though less potent inducers of MCP-1 expression.[25–27] IFN$\gamma$ was recently shown to induce MCP-1 in human microvascular EC.[46] M-CSF was reported to induce MCP-1, though we did not detect the M-CSF receptor c-fms in EC by Northern analysis.[47] Given the role that lipids and monocytes play in the natural history of atherosclerosis, it is of interest that minimally modified LDL induce MCP-1 production in EC and smooth muscle cells.[48] Thrombin was recently found to induce expression of MCP-1 in monocytes and, less prominently, in EC.[49] The C–C chemokine RANTES was produced by EC exposed to TNF and IFN$\gamma$.[50]

The molecular basis of stimulation of chemokine expression in EC has been studied to a limited extent. Induction by inflammatory signals and thrombin is protein synthesis independent in EC, but, interestingly, not in monocytes.[49] Direct demonstration of enhanced gene transcription was obtained for MCP-1 and IL-8 by nuclear run off analysis.[20,21]

*In vivo studies:* EC at sites of delayed type hypersensitivity reactions and kidney allograft rejection express the C–C chemokine RANTES.[51,52] *In vivo* studies on chemokines in vessel wall pathology have largely been restricted to atherosclerosis. MCP-1 expression has been detected in atheromatous lesions of rabbits, primates and man.[53–56] IL-8 and MCP-1 mRNA have been detected in increased amounts of aortic aneurisms.[57] Chemokine gene expression has been detected in various cellular elements, including smooth muscle cells, EC and mononuclear phagocytes, with somewhat different results in different studies. In the only study with mAb,[53] cell populations positive for MCP-1 were different in lesions representative of different stages of the natural history of atherosclerosis. EC staining was prominent in diffuse intimal thickening and in fatty streaks, whereas it was weak in atheromatous lesions. Subendothelial macrophages were strongly positive for MCP-1 in fatty streak lesions and in atherosclerotic plaques. In plaques, a few intimal smooth muscle cells stained for MCP-1. These results suggest that EC and macrophages are the major source of MCP-1 in early atherosclerotic lesions.[53] Monocyte adhesion and infiltration is an early event in the natural history of atherosclerosis.[13,11] Mononuclear phagocyte infiltration is also a prominent feature of vasculitis.[58] Locally produced MCP-1 may play an important role in regulating extravasation of leukocytes, of monocytes in particular, in vessel wall pathology.

### Endothelial adhesive molecules

The recruitment of leukocytes into sites of inflammation involves a cascade of sequential events controlled by the interaction between adhesion molecules expressed by leukocytes and by the endothelium. This multi-step process is a cell to cell adhesive reaction that involves specific binding of membrane receptors on one cell to counter-receptor structures on the other cell. Several adhesion receptors belonging to different families including integrins, selectins, and immunoglobulin-like molecules have been shown to participate in this mechanism.[1,2] In the current model, selectins are implicated in the initial rolling, while adhesion receptors from the integrin family and the immunoglobulin superfamily are involved in the firm attachment, flattening and extravasation of leukocytes.[1,2,59,60] Leukocytes have to adhere to the endothelium before transmigrating, and it is difficult to distinguish between adhesion molecules involved only in adherence and proteins involved in the transmigration process. However, several studies have shown that intercellular cell adhesion molecule 1 (ICAM-1), vascular cell adhesion molecule 1 (VCAM-1), platelet endothelial cell adhesion molecule 1 (PECAM-1) and selectins are important for leukocyte diapedesis between endothelial cells.

*Selectins:* The selectin family comprises three proteins: E-selectin (CD62E), L-selectin (CD62L) and P-selectin (CD62P).[59,61] They all contain a lectin domain, an epidermal growth factor domain, and a variable number of short consensus repeats of 60 amino acids present in the complement regulatory proteins. The three selectin genes are located on chromosome 1.[62,63]

E- and P-selectins are expressed on endothelial cells, while L-selectin expression is restricted to leukocytes. Selectins bind to carbohydrate ligands via their lectin domains. It has been shown that tetrasaccharides sialyl Lewis X and sialyl Lewis A (sLeX, sLeA) have a ligand activity for all the three selectins.[61,64] The role of selectins in leukocyte transmigration is still debated.

E-selectin. E-selectin (CD62E) is a 115 kDa glycoprotein, only expressed on EC after activation by IL-1, TNF-α,[65] or bacterial endotoxin such as LPS.[66] After EC stimulation, newly synthesized E-selectin is rapidly detected with a maximal surface expression after 3–6 h and a return to basal levels within 24 h.[65,67] This rapid down-regulation, although not completely understood, has been explained by the release of a soluble form of E-selectin,[68,69] and internalization of the molecule.[70] This regulation of E-selectin expression might be crucial to control leukocyte accumulation in inflammatory responses. Several ligands for E-selectin have been identified on leukocytes, but have not yet been cloned.[71,72] Monoclonal antibodies specific for E-selectin have been shown to inhibit leukocyte transmigration.[73–76] It has been suggested that binding of leukocytes to E-selectin on activated endothelium upregulates CD11b (Mac-1) on the leukocytes, and induces an increased adhesion through an ICAM-1/Mac-1 interaction.[73,77,78]

P-selectin. P-selectin (CD62P), previously termed PADGEM or GMP-140, is a single-chain glycoprotein of 140 kDa, expressed in platelets and endothelial cells. In platelets, P-selectin is stored in α-granules,[79] whereas in endothelial cells it is found in Weibel–Palade bodies.[80,81] After activation, P-selectin is mobilized to the external plasma membrane within minutes. This increase in P-selectin expression is transient, and the protein is rapidly internalized inside the cell, where it is degraded or recycled.[82,83] P-selectin is also upregulated transcriptionally by TNF-α.[67] Two ligands has been identified: the P-selectin glycoprotein ligand-1 (PSGL-1), expressed on various leukocytes[84] and a 120 kDa ligand expressed only on myeloid cells.[85] P-selectin deficient mice have been shown to be deficient in leukocyte extravasation.[60]

*IgG superfamily:*

ICAM-1. In general, interaction of leukocytes with ICAM-1 seems to be necessary for their extravasation. ICAM-1 (CD54) is a single chain membrane glycoprotein of 80–115 kDa, with five Ig-like repeats in its extracellular domain.[86] ICAM-1 is moderately expressed on resting endothelial cells, but release of cytokines at sites of inflammation and immune response such as TNF-α, IL-1 or IFNγ results in augmented cellular expression of ICAM-1.[87,88] The expression of ICAM-1 has also been demonstrated on lymphocytes, monocytes and other non-haematopoietic cells, like fibroblasts, epithelial cells and mucosal cells.[87,89] ICAM-1 is a ligand for CD11a/CD18 (LFA-1)[90] and for CD11b/CD18 (Mac-1).[91] The primary binding site for CD11a/CD18 is located in the $NH_2$-terminal first Ig-like domain of ICAM-1, with domain 2 also involved in this interaction,[92] while the one for CD11b/CD18 is localized to the third Ig-like domain.[93] ICAM-1 is also a receptor for the major group of rhinoviruses[94,95] and the malaria trophozoite *Plasmodium falciparum*,[96] the binding site for both ligands, though distinct from the LFA-1 binding site, is located in the first two domains of the ICAM-1 molecule.[92,97] In addition, ICAM-1 is a receptor for CD43[98] and hyaluronan.[99] The interaction between Mac-1/LFA-1 (CD11a,b/CD18) and endothelial ICAM-1 is a well documented adhesion pathway, important in the adhesion and extravasation of leukocytes.[73,74,100–102] It has been shown recently that fibrinogen (Fg) is a ligand for ICAM-1, and that Fg binding to ICAM-1 results in enhanced adhesion of leukocytes to EC monolayers,[103] and an increase in their transendothelial migration.[104] These results suggest that Fg must act as a bridging molecule: for monocyte and polymorphonuclear leukocyte adhesion, it could bind to leukocyte CD11b/CD18[105] and to endothelial cell ICAM-1, while for lymphocyte adhesion it could interact with two ICAM-1 molecules on opposing cells. This interaction between Fg and ICAM-1 was inhibited by a commercially available mAb specific for ICAM-1, LB2, epitope-mapped to the first immunoglobulin domain of ICAM-1,[97] suggesting that this domain is involved in the Fg interaction with ICAM-1. However, the inhibition obtained with LB2 on Fg-dependent adhesion was only partial, even at a high mAb concentration. This limited inhibition might reflect the fact that LB2 is not reacting with the exact binding site of Fg on ICAM-1, but rather with a nearby site. Alternatively, an unidentified Fg receptor present at the surface of endothelial cells could contribute to Fg-mediated adhesion of leukocytes. Both Fg-mediated leukocyte adhesion and transendothelial migration could be inhibited by a peptide from the fibrinogen γ chain.[106]

VCAM-1. VCAM-1 (CD106) is a transmembrane glycoprotein of 110 kDa expressed only on cytokine-activated endothelium.[107,108] A protein containing six Ig-like domains was initially cloned (6D VCAM-1),[109] but this form arises from an alternative splicing of a seven Ig-like domain of VCAM-1 (7D VCAM-1), which is the dominant form on activated endothelium.[110–112] VCAM-1 is a ligand for α4β1 (VLA-4) and α4β7 integrins.[113–116] VLA-4 binds VCAM-1 through the first and the fourth Ig domain.[117,118] Using monoclonal antibodies, several studies have shown that VCAM-1 is involved in the transmigration of monocytes and eosinophils,[74,75] but its involvement in lymphocyte transendothelial migration remains to be clarified.[119,120]

PECAM-1. PECAM-1 (CD31) is a 130 kDa glycoprotein expressed on endothelial cells, platelets and some leukocytes.[121] CD31 is constitutively expressed on endothelial cells, and its expression is not increased by cytokines.[122] Molecular cloning studies have shown that CD31 is composed of six extracellular Ig-like domains, a short transmembrane region, and a relatively long cytoplasmic tail of 118 amino acid-containing potential sites for posttranslational modifications.[122–125] Alternative splicing of the cytoplasmic tail can generate multiple CD31 isoforms that may regulate phosphorylation, cytoskeletal association and ligand affinity of the protein.[126] CD31 is heavily glycosylated and glycosylation accounts for 40% of the mass of CD31.[123] PECAM-1 appears to be able to interact both with itself in a homophylic interaction and with other molecules in a heterophylic interaction.[125,127] In endothelial cells, CD31 is localized at intercellular junctions,[122,125] and plays an important role in adhesion of endothelial cells.[125] The high level of constitutive expression of PECAM-1 in endothelial cells suggests that its function might be regulated, and phosphorylation of the cytoplasmic domain has been demonstrated.[128] PECAM-1 is directly involved in the process of leukocyte diapedesis between endothelial cells, as demonstrated by inhibition studies using anti-PECAM-1 monoclonal antibodies and soluble recombinant PECAM-1.[129] Leukocytes blocked in transmigration by anti-PECAM-1 antibodies remained attached to the endothelium, clearly implicating PECAM-1 in diapedesis rather than in adhesion.[129]

### Regulation of endothelial cell-to-cell junctions

Circulating cells infiltrate into tissues migrating through intercellular junctions. These organelles are formed by a complex network of transmembrane proteins linked to a well developed plasmalemmal undercoat.[130–132] One of the typical characteristics of endothelial junctions is their dynamic organization. Endothelial cells are able to rapidly change the architecture of the junctions to allow the passage of circulating blood cells. This effect, in most cases, is quickly reversible and the endothelium is able to disorganize/reorganize its intercellular junctions within minutes. Interendothelial junctions present a different degree of complexity along the vascular tree responding to different functional requirements.[133] For instance, they are well organized and numerous in large arteries or in the blood vessels of the brain where the control of permeability must be strict, whereas they are very primitive in the post-capillary venules, where cell extravasation and exchange of plasma constituents need to be particularly efficient.[133]

On the basis of morphological and functional characteristics at least four types of junctions have been described in endothelial cells. These are: tight junctions (TJ),[134–136] adherence junctions (AJ),[132] gap junctions[137] and syndesmos.[138]

Although there is a great deal of information regarding the molecules that mediate leukocyte adhesion to the endothelium, the mechanisms by which leukocytes trigger the opening of endothelial cell junctions is still obscure. In many conditions the passage of leukocytes through endothelial junctions is a non-toxic process that does not increase endothelial permeability *per se* or cause vascular damage.[139]

Chemoattractants and adhesive molecules stimulate neutrophils to secrete oxygen free radicals, lipid metabolites and proteases, each of which is a potential agonist of endothelial permeability.[131] However, experimental evidence suggests that these reactive agents are not necessary for neutrophil extravasation.

In patients with chronic granulomatous disease leukocytes are unable to make oxygen metabolites but can extravasate and infiltrate in areas of inflammation and form pus.[139]

Inhibitors of proteases do not affect neutrophil extravasation in different experimental conditions.[139] In addition, the possibility that leukocyte passage through the endothelium requires protease digestion of membrane proteins seems unlikely in view of the very rapid, within seconds, closure and reorganization of the junctions after leukocyte diapedesis.

These observations, however, do not exclude the possibility that oxygen free radicals and proteases might act as contributing factors in leukocyte extravasation, inducing endothelial cell damage and mediating oedema during sustained inflammatory reactions.

The question of how leukocytes pass through endothelial clefts remains open. An interesting possibility is that leukocyte adhesion to endothelial cells could cause a cascade of events that resembles that induced by soluble agonists of endothelial permeability. In particular, leukocyte ligation to endothelial adhesive molecules (such as selectins, VCAM or ICAM-1) could generate intracellular signals similar to those induced by permeability increasing agents. It has been found[140] that endothelial cells respond to neutrophil contact and migration by increasing intracellular calcium. Similarly, inhibitors of intracellular $Ca^{2+}$ block neutrophil transmigration. In addition, ICAM-1 activation by specific antibodies leads to cortactin phosphorylation.[141] This or other signals could induce changes in cleft molecular organization (see above), leading to the opening of gaps between endothelial cells. According to this hypothesis endothelial cells would not only play an important role in regulating leukocyte attachment to their surface but also actively modulate their extravasation.

Leukocytes might find preferential pathways for their passage through the interendothelial clefts. As discussed above, TJ and AJ comprise a system of discrete ion selective pores rather than an absolute seal around the cells.[134] In endothelial cells, the presence of areas of junctionless clefts that regulate the transendothelial transport of high molecular weight proteins has been described.[142] Leukocytes might be directed to these pores through the concentration gradient of specific adhesive molecules such as PECAM. Their passage would require them to squeeze through the pores accompanied by a rearrangement of the endothelial cell cytoskeleton organization around these structures.

There might be differences comparing the modalities of leukocyte extravasation for different types of vessels, for example lymphatic versus large vessels, where the clefts present different levels of complexity. There might also be distinct mechanisms regulating the passage of the different types of leukocytes or of other types of cells.

### Concluding remarks

We begin now to understand that leukocytes and endothelial cells are able to communicate and reciprocally modulate their responses. Following inflammatory stimulation endothelial cells attract and localize leukocytes through the release of chemokines and expression of adhesive molecules. Leukocytes in turn might transfer signals to the endothelium releasing soluble mediators, such as cytokines, oxidation products and lytic enzymes. Adhesion of leukocytes to endothelial adhesive molecules might also cause endothelial cell activation facilitating leukocyte passage through interendothelial clefts. Leukocyte extravasation is not always accompanied by endothelial cell damage and an increase in permeability. In contrast, it seems that the opening of endothelial junctions is a well regulated process that is frequently reversible. Future work is required to fully understand how we might modulate the cross-talk between endothelial cells and leukocytes. This appears to be a difficult task considering the complexity and the number of soluble and membrane bound molecules which involved interplay.

### References

1. Carlos TM, Harlan JM. Leukocyte-endothelial adhesion molecules. *Blood* 1994; **84:** 2068–2101.
2. Springer TA. Traffic signals for lymphocyte recirculation and leukocyte emigration: the multistep paradigm. *Cell* 1994; **76:** 301–314.
3. Luscinskas FW, Lawler J. Integrins as dynamic regulators of vascular function. *FASEB J* 1994; **8:** 929–938.
4. Imhof BA, Dunon D. Leukocyte migration and adhesion. *Adv Immunol* 1995; **58:** 345–416.
5. Mantovani A, Bussolino F, Dejana E. Cytokine regulation of endothelial cell function. *FASEB J* 1992; **6:** 2591–2599.
6. Dejana E, Corada M, Lampugnani MG. Endothelial cell-to-cell junctions. *FASEB J* 1995; **9:** 910–918.
7. Rosenkranz Weiss P, Sessa WC, Milstien S, Kaufman S, Watson CA, Pober JS. Regulation of nitric oxide synthesis by proinflammatory cytokines in human umbilical vein endothelial cells. Elevations in tetrahydrobiopterin levels enhance endothelial nitric oxide synthase specific activity. *J Clin Invest* 1994; **93:** 2236–2243.
8. Rossi V, Breviario F, Ghezzi P, Dejana E, Mantovani A. Prostacyclin synthesis induced in vascular cells by interleukin-1. *Science* 1985; **229:** 174–176.
9. Lampugnani MG, Resnati M, Raiteri M, *et al.* A novel endothelial specific membrane protein is a marker of cell–cell contacts. *J Cell Biol* 1992; **118:** 1511–1522.
10. Pober J, Cotran RS. Cytokines and endothelial cell biology. *Physiol Rev* 1990; **70:** 427–451.
11. Ross R. The pathogenesis of atherosclerosis: a perspective for the 1990s. *Nature* 1993; **362:** 801–809.
12. Mantovani A, Dejana E. Cytokines as communication signals between leukocytes and endothelial cells. *Immunol Today* 1989; **10:** 370–375.
13. Libby P, Hansson GK. Biology of disease. Involvement of the immune system in human atherogenesis: current knowledge and unanswered questions. *Lab Invest* 1991; **64:** 5–15.
14. Baggiolini M, Dewald B, Moser B. Interleukin-8 related chemotactic cytokines-CXC and CC chemokines. *Adv Immunol* 1994; **55:** 97–179.
15. Oppenheim JJ, Zachariae COC, Mukaida N, Matsushima K. Properties of the novel proinflammatory supergene intercrine cytokine family. *Annu Rev Immunol* 1991; **9:** 617–648.
16. Dixit VM, Green S, Sarma V, *et al.* Tumor necrosis factor-alpha induction of novel gene products in human endothelial cells including a macrophage-specific chemotaxin. *J Biol Chem* 1990; **265:** 2973–2978.
17. Wen D, Rowland A, Derynck R. Expression and secretion of gro/MGSA by stimulated human endothelial cells. *EMBO J* 1989; **8:** 1761–1766.
18. Strieter RM, Kunkel SL, Showell HJ, *et al.* Endothelial cell gene expression of a neutrophil chemotactic factor by TNF-alpha, LPS, and IL-1 beta. *Science* 1989; **243:** 1467–1469.
19. Schroder JM, Christophers E. Secretion of novel and homologous neutrophil-activating peptides by LPS-stimulated human endothelial cells. *J Immunol* 1989; **142:** 244–251.
20. Sica A, Matsushima K, Van Damme J, *et al.* IL-1 transcriptionally activates the neutrophil chemotactic factor/IL-8 gene in endothelial cells. *Immunology* 1990; **69:** 548–553.
21. Sica A, Wang JM, Colotta F, *et al.* Monocyte chemotactic and activating factor gene expression induced in endothelial cells by IL-1 and tumor necrosis factor. *J Immunol* 1990; **144:** 3034–3038.
22. Rollins BJ, Yoshimura T, Leonard EJ, Pober JS. Cytokine-activated human endothelial cells synthesize and secrete a monocyte chemoattractant, MCP-1/JE. *Am J Pathol* 1990; **136:** 1229–1233.
23. Gimbrone MAJ, Obin MS, Brock AF, *et al.* Endothelial interleukin-8: a novel inhibitor of leukocyte–endothelial interactions. *Science* 1989; **246:** 1601–1603.

24. Sironi M, Sciacca FL, Matteucci C, *et al.* Regulation of endothelial and mesothelial cell function by interleukin-13: selective induction of vascular cell adhesion molecule-1 and amplification of interleukin-6 production. *Blood* 1994; **84:** 1913–1921.
25. Rollins BJ, Pober JS. Interleukin-4 induces the synthesis and secretion of MCP-1JE by human endothelial cells. *Am J Pathol* 1991; **138:** 1315–1319.
26. Howells G, Pham P, Taylor D, Foxwell B, Feldmann M. Interleukin 4 induces interleukin 6 production by endothelial cells: synergy with interferon-gamma. *Eur J Immunol* 1991; **21:** 97–101.
27. Colotta F, Sironi M, Borre A, Luini W, Maddalena F, Mantovani A. Interleukin 4 amplifies monocyte chemotactic protein and interleukin 6 production by endothelial cells. *Cytokine* 1992; **4:** 4–28.
28. Korpelainen EI, Gamble JR, Smith WB, *et al.* The receptor for interleukin 3 is selectively induced in human endothelial cells by tumor necrosis factor alpha and potentiates interleukin 8 secretion and neutrophil transmigration. *Proc Natl Acad Sci USA* 1993; **90:** 11137–11141.
29. Jeannin P, Delneste Y, Gosset P, *et al.* Histamine induces interleukin-8 secretion by endothelial cells. *Blood* 1994; **84:** 2229–2233.
30. Karakurum M, Shreeniwas R, Chen J *et al.* Hypoxic induction of interleukin-8 gene expression in human endothelial cells. *Blood* 1993; **81:** 2492–2495.
31. Karakurum M, Shreeniwas R, Chen J, *et al.* Hypoxic induction of interleukin-8 gene expression in human endothelial cells. *J Clin Invest* 1994; **93:** 1564–1570.
32. Kaplanski G, Porat R, Aiura K, Erban JK, Gelfand JA, Dinarello CA. Activated platelets induce endothelial secretion of interleukin-8 in vitro via an interleukin-1-mediated event. *Blood* 1993; **81:** 2492–2495.
33. Herbert CA, Luscinskas FW, Kiely JM, *et al.* Endothelial and leukocyte forms of IL-8. Conversion by thrombin and interactions with neutrophils. *J Immunol* 1990; **145:** 3033–3040.
34. Carveth HJ, Bohnsack JF, McIntyre TM, Baggiolini M, Prescott SM, Zimmerman GA. Neutrophil activating factor (NAF) induces polymorphonuclear leukocyte adherence to endothelial cells and to subendothelial matrix proteins. *Biochem Biophys Res Commun* 1989; **162:** 387–393.
35. Hechtman DH, Cybulsky MI, Fuchs HJ, Baker JB, Gimbrone MAJ. Intravascular IL-8. Inhibitor of polymorphonuclear leukocyte accumulation at sites of acute inflammation. *J Immunol* 1991; **147:** 883–892.
36. Ley K, Baker JB, Cybulsky MI, Gimbrone MA, Luscinskas FW. Intravenous interleukin-8 inhibits granulocyte emigration from rabbit mesenteric venules without altering L-selectin expression or leukocyte rolling. *J Immunol* 1993; **151:** 6347–6357.
37. Huber AR, Kunkel SL, Todd RF, Weiss SJ. Regulation of transendothelial neutrophil migration by endogenous interleukin-8. *Science* 1991; **254:** 99–102.
38. Smith WB, Gamble JR, Clarklewis I, Vadas MA. Chemotactic desensitization of neutrophils demonstrates interleukin-8 (IL-8)-dependent and IL-8-independent mechanisms of transmigration through cytokine-activated endothelium. *Immunology* 1993; **78:** 491–497.
39. Rot A. Endothelial cell binding of NAP-1/IL-8: role in neutrophil emigration. *Immunol Today* 1992; **13:** 291–294.
40. Hadley TJ, Lu ZH, Wasniowska K, *et al.* Postcapillary venule endothelial cells in kidney express a multispecific chemokine receptor that is structurally and functionally identical to the erythroid isoform, which is the Duffy blood group antigen. *J Clin Invest* 1994; **94:** 985–991.
41. Peiper SC, Wang ZX, Neote K, *et al.* The Duffy antigen receptor for chemokines (DARC) is expressed in endothelial cells of Duffy negative individuals who lack the erythrocyte receptor. *J Exp Med* 1995; **181:** 1311–1317.
42. Wang JM, Taraboletti G, Matsushima K, Van Damme J, Mantovani A. Induction of haptotactic migration of melanoma cells by neutrophil activating protein/interleukin-8. *Biochem Biophys Res Commun* 1990; **169:** 165–170.
43. Schwartz D, Andalibi A, Chaverrialmada L, *et al.* Role of the GRO family of chemokines in monocyte adhesion to MM-LDL-stimulated endothelium. *J Clin Invest* 1994; **94:** 1968–1973.
44. Narumi S, Wyner LM, Stoler MH, Tannenbaum CS, Hamilton TA. Tissue-specific expression of murine IP-10 mRNA following systemic treatment with interferon-gamma. *J Leukoc Biol* 1992; **52:** 27–33.
45. Gómez-Chiarri M, Hamilton TA, Egido J, Emancipator SN. Expression of IP-10, a lipopolysaccharide- and interferon-gamma-inducible protein, in murine mesangial cells in culture. *Am J Pathol* 1993; **142:** 433–439.
46. Brown Z, Gerritsen ME, Carley WW, Strieter RM, Kunkel SL, Westwick J. Chemokine gene expression and secretion by cytokine-activated human microvascular endothelial cells – differential regulation of monocyte chemoattractant protein-1 and interleukin-8 in response to interferon-gamma. *Am J Pathol* 1994; **145:** 913–921.
47. Shyy YJ, Wickham LL, Hagan JP, *et al.* Human monocyte colony-stimulating factor stimulates the gene expression of monocyte chemotactic protein-1 and increases the adhesion of monocytes to endothelial monolayers. *J Clin Invest* 1993; **92:** 1745–1751.
48. Cushing SD, Berliner JA, Valente AJ, *et al.* Minimally modified low density lipoprotein induces monocyte chemotactic protein 1 in human endothelial cells and smooth muscle cells. *Proc Natl Acad Sci USA* 1990; **87:** 5134–5138.
49. Colotta F, Sciacca FL, Sironi M, Luini W, Rabiet MJ, Mantovani A. Expression of monocyte chemotactic protein-1 by monocytes and endothelial cells exposed to thrombin. *Am J Pathol* 1994; **144:** 975–985.
50. Marfaingkoka A, Devergne O, Gorgone G, *et al.* Regulation of the production of the RANTES chemokine by endothelial cells—synergistic induction by IFN-gamma plus TNF-alpha and inhibition by IL-4 and IL-13. *J Immunol* 1995; **154:** 1870–1878.
51. Devergne O, Marfaing-Koka A, Schall TJ, *et al.* Production of the RANTES chemokine in delayed-type hypersensitivity reactions: involvement of macrophages and endothelial cells. *J Exp Med* 1994; **179:** 1689–1694.
52. Pattison J, Nelson PJ, Huie P, *et al.* RANTES chemokine expression in cell-mediated transplant rejection of the kidney. *Lancet* 1994; **343:** 209–211.
53. Takeya M, Yoshimura T, Leonard EJ, Takahashi K. Detection of monocyte chemoattractant protein-1 in human atherosclerotic lesions by an anti-monocyte chemoattractant protein-1 monoclonal antibody. *Hum Pathol* 1993; **24:** 534–539.
54. Yu X, Dluz S, Graves DT, *et al.* Elevated expression of monocyte chemoattractant protein 1 by vascular smooth muscle cells in hypercholesterolemic proteins. *Proc Natl Acad Sci USA* 1992; **89:** 6953–6957.
55. Yla Herttuala S, Lipton BA, Rosenfeld ME, *et al.* Expression of monocyte chemoattractant protein 1 in macrophage-rich areas of human and rabbit atherosclerotic lesions. *Proc Natl Acad Sci USA* 1991; **88:** 5252–5256.
56. Nelken NA, Coughlin SR, Gordon D, Wilcox JN. Monocyte chemoattractant protein-1 in human atheromatous plaques. *J Clin Invest* 1991; **88:** 1121–1127.
57. Koch AE, Kunkel SL, Pearce WH, *et al.* Enhanced production of the chemotactic cytokines interleukin-8 and monocyte chemoattractant protein-1 in human abdominal aortic aneurysms. *Am J Pathol* 1993; **142:** 1423–1431.
58. Fauci AS, Haynes BF, Katz P. The spectrum of vasculitis. *Ann Intern Med* 1978; **89** (Part 1): 660–676.
59. Bevilacqua MP, Nelson RM. Selectins. *J Clin Invest* 1993; **91:** 379–387.
60. Mayadas TN, Johnson RC, Rayburn H, Hynes RO, Wagner DD. Leukocyte rolling and extravasation are severely compromised in P selectin-deficient mice. *Cell* 1993; **74:** 541–554.
61. Lasky LA. Selectins: interpreters of cell-specific carbohydrate information during inflammation. *Science* 1992; **258:** 964–969.
62. Watson ML, Kingsmore SF, Johnston GI, *et al.* Genomic organization of the selectin family of leukocyte adhesion molecules on human and mouse chromosome 1. *J Exp Med* 1990; **172:** 263–272.
63. Collins T, Williams A, Johnston GI, *et al.* Structure and chromosomal location of the gene for endothelial-leukocyte adhesion molecule 1. *J Biol Chem* 1991; **266:** 2466–2473.
64. Springer TA, Lasky LA. Sticky sugars for selectins. *Nature* 1991; **349:** 196–197.
65. Fries JW, Williams AJ, Atkins RC, Newman W, Lipscomb MF, Collins T. Expression of VCAM-1 and E-selectin in an *in vivo* model of endothelial activation. *Am J Pathol* 1993; **143:** 725–737.
66. Shimizu Y, Newman W, Gopal TV, *et al.* Four molecular pathways of T cell adhesion to endothelial cells: roles of LFA-1, VCAM-1, and ELAM-1 and changes in pathway hierarchy under different activation conditions. *J Cell Biol* 1991; **113:** 1203–1212.
67. Weller A, Isenmann S, Vestweber D. Cloning of the mouse endothelial selectins. Expression of both E- and P-selectin is inducible by tumor necrosis factor alpha. *J Biol Chem* 1992; **267:** 15176–15183.
68. Leeuwenberg JF, Smeets EF, Neefjes JJ, *et al.* E-selectin and intercellular adhesion molecule-1 are released by activated human endothelial cells *in vitro*. *Immunology* 1992; **77:** 543–549.
69. Newman W, Beall LD, Carson CW, *et al.* Soluble E-selectin is found in supernatants of activated endothelial cells and is elevated in the serum of patients with septic shock. *J Immunol* 1993; **150:** 644–654.
70. von Asmuth EJ, Smeets EF, Ginsel LA, Onderwater JJ, Leeuwenberg JF, Buurman WA. Evidence for endocytosis of E-selectin in human endothelial cells. *Eur J Immunol* 1992; **22:** 2519–2526.
71. Ohmori K, Takada A, Yoneda T, *et al.* Differentiation-dependent expression of sialyl stage-specific embryonic antigen-1 and I-antigens on human lymphoid cells and its implications for carbohydrate-mediated adhesion to vascular endothelium. *Blood* 1993; **81:** 101–111.
72. Berg EL, Yoshino T, Rott LS, *et al.* The cutaneous lymphocyte antigen is a skin lymphocyte homing receptor for the vascular lectin endothelial cell-leukocyte adhesion molecule 1. *J Exp Med* 1991; **174:** 1461–1466.
73. Luscinskas FW, Cybulsky MI, Kiely JM, Peckins CS, Davis VM, Gimbrone MA Jr. Cytokine-activated human endothelial monolayers support enhanced neutrophil transmigration via a mechanism involving both endothelial-leukocyte adhesion molecule-1 and intercellular adhesion molecule-1. *J Immunol* 1991; **146:** 1617–1625.
74. Hakkert BC, Kuijpers TW, Leeuwenberg JF, van Mourik JA, Roos D. Neutrophil and monocyte adherence to and migration across monolayers of cytokine-activated endothelial cells: the contribution of CD18, ELAM-1, and VLA-4. *Blood* 1991; **78:** 2721–2726.
75. Meerschaert J, Furie MB. Monocytes use either CD11/CD18 or VLA-4 to migrate across human endothelium *in vitro*. *J Immunol* 1994; **152:** 1915–1926.

76. Ebisawa M, Bochner BS, Georas SN, Schleimer RP. Eosinophil transendothelial migration induced by cytokines. I. Role of endothelial and eosinophil adhesion molecules in IL-1 beta-induced transendothelial migration. *J Immunol* 1992; **149:** 4021–4028.
77. Kuijpers TW, Hoogerwerf M, Roos D. Neutrophil migration across monolayers of resting or cytokine-activated endothelial cells: role of intracellular calcium changes and fusion of specific granules with the plasma membrane. *J Immunol* 1992; **148:** 72–77.
78. Hakkert BC, Rentenaar JM, van Aken WG, Roos D, Mourik JA. A three dimensional model system to study the interaction between human leukocytes and endothelial cells. *Eur J Immunol* 1990; **20:** 2775–2781.
79. Stenberg PE, McEver RP, Shuman MA, Jacques YV, Bainton DF. A platelet alpha-granule membrane protein (GMP-140) is expressed on the plasme membrane after activation. *J Cell Biol* 1985; **101:** 880–886.
80. McEver RP, Beckstead JH, Moore KL, Marshall-Carlson L, Bainton DF. GMP-140, a platelet alpha-granule membrane protein, is also synthesized by vascular endothelial cells and is localized in Weibel–Palade bodies. *J Clin Invest* 1989; **84:** 92–99.
81. Bonfanti R, Furie BC, Furie B, Wagner DD. PADGEM (GMP-140) is a component of Weibel–Palade bodies of human endothelial cells. *Blood* 1989; **73:** 1109–1112.
82. Fujimoto T, McEver RP. The cytoplasmic domain of P-selectin is phosphorylated on serine and threonine residues. *Blood* 1993; **82:** 1758–1766.
83. Green SA, Setiadi H, McEver RP, Kelly RB. The cytoplasmic domain of P-selectin contains a sorting determinant that mediates rapid degradation in lysosomes. *J Cell Biol* 1994; **124:** 435–448.
84. Sako D, Chang XJ, Barone KM, *et al.* Expression cloning of a functional glycoprotein ligand for P-selectin. *Cell* 1993; **75:** 1179–1186.
85. Moore KL, Eaton SF, Lyons DE, Lichenstein HS, Cummings RD, McEver RP. The P-selectin glycoprotein ligand for human neutrophils displays sialylated, fucosylated, *O*-linked poly-*N*-acetyllactosamine. *J Biol Chem* 1994; **269:** 23318–23327.
86. Simmons D, Makgoba MW, Seed B. ICAM, an adhesion ligand of LFA-1, is homologous to the neural cell adhesion molecule NCAM. *Nature* 1988; **331:** 624–627.
87. Dustin ML, Rothlein R, Bhan AK, Dinarello CA, Springer TA. Induction of IL 1 and interferon-gamma: tissue distribution, biochemistry, and function of a natural adherence molecule (ICAM-1). *J Immunol* 1986; **137:** 245–254.
88. Pober JS, Gimbrone MA, Lapierre LA, *et al.* Overlapping patterns of activation of human endothelial cells by interleukin 1, tumor necrosis factor and immune interferon. *J Immunol* 1986; **137:** 1893–1896.
89. Schulz TF, Mitterer M, Vogetseder W, Bock G, Myones BL, Dierich MP. Identification and characterization of a novel membrane activation antigen with wide cellular distribution. *Eur J Immunol* 1988; **18:** 7–11.
90. Marlin SD, Springer TA. Purified intercellular adhesion molecule-1 (ICAM-1) is a ligand for lymphocyte function-associated antigen 1 (LFA-1). *Cell* 1987; **51:** 813–819.
91. Diamond MS, Staunton DE, de Fougerolles AR, *et al.* ICAM-1 (CD54): a counter-receptor for Mac-1 (CD11b/CD18). *J Cell Biol* 1990; **111:** 3129–3139.
92. Staunton DE, Dustin ML, Erickson HP, Springer TA. The arrangement of the immunoglobulin-like domains of ICAM-1 and the binding sites for LFA-1 and rhinovirus. *Cell* 1990; **61:** 243–254.
93. Diamond MS, Staunton DE, Marlin SD, Springer TA. Binding of the integrin Mac-1 (CD11b/CD18) to the third immunoglobulin-like domain of ICAM-1 (CD54) and its regulation by glycosylation. *Cell* 1991; **65:** 961–971.
94. Staunton DE, Merluzzi VJ, Rothlein R, Barton R, Marlin SD, Springer TA. A cell adhesion molecule, ICAM-1, is the major surface receptor for rhinoviruses. *Cell* 1989; **56:** 849–853.
95. Greve JM, Davis G, Meyer AM, *et al.* The major human rhinovirus receptor is ICAM-1. *Cell* 1989; **56:** 839–847.
96. Berendt AR, Simmons DL, Tansey J, Newbold CI, Marsh K. Intercellular adhesion molecule-1 is an endothelial cell adhesion receptor for *Plasmodium falciparum*. *Nature* 1989; **341:** 57–59.
97. Berendt AR, McDowall A, Craig AG, *et al.* The binding site on ICAM-1 for *Plasmodium falciparum*-infected erythrocytes overlaps, but is distinct from, the LFA-1-binding site. *Cell* 1992; **68:** 71–81.
98. Rosenstein Y, Park JK, Hahn WC, Rosen FS, Bierer BE, Burakoff SJ. CD43, a molecule defective in Wiskott–Aldrich syndrome, binds ICAM-1. *Nature* 1991; **354:** 233–235.
99. McCourt PAG, Ek B, Forsberg N, Gustafson S. Intercellular adhesion molecule-1 is a cell surface receptor for hyaluronan. *J Biol Chem* 1994; **269:** 30081–30084.
100. Smith CW, Marlin SD, Rothlein R, Toman C, Anderson DC. Cooperative interactions of LFA-1 and Mac-1 with intercellular adhesion molecule-1 in facilitating adherence and transendothelial migration of human neutrophils *in vitro*. *J Clin Invest* 1989; **83:** 2008–2017.
101. Furie MB, Tancinco MC, Smith CW. Monoclonal antibodies to leukocyte integrins CD11a/CD18 and CD11b/CD18 or intercellular adhesion molecule-1 inhibit chemoattactant-stimulated neutrophil transendothelial migration *in vitro*. *Blood* 1991; **78:** 2089–2097.
102. Anderson DC, Rothlein R, Marlin SD, Krater SS, Smith CW. Impaired transendothelial migration by neonatal neutrophils: abnormalities of Mac-1 (CD11b/CD18)-dependent adherence reactions. *Blood* 1990; **76:** 2613–2621.
103. Languino LR, Plescia J, Duperray A, *et al.* Fibrinogen mediates leukocyte adhesion to vascular endothelium through an ICAM-1-dependent pathway. *Cell* 1993; **73:** 1423–1434.
104. Languino LR, Duperray A, Joganic KJ, Fornaro M, Thornton GB, Altieri DC. Regulation of leukocyte–endothelium interaction and leukocyte transendothelial migration by intercellular adhesion molecule 1–fibrinogen recognition. *Proc Natl Acad Sci USA* 1995; **92:** 1505–1509.
105. Altieri DC, Bader R, Mannucci PM, Edgington TS. Oligospecificity of the cellular adhesion receptor Mac-1 encompasses an inducible recognition for fibrinogen. *J Cell Biol* 1988; **107:** 1893–1900.
106. Altieri DC, Duperray A, Plescia J, Thornton GB, Languino LR. Structural recognition of a novel fibrinogen gamma chain sequence (117–133) by intercellular adhesion molecule-1 mediates leukocyte–endothelium interaction. *J Biol Chem* 1995; **270:** 696–699.
107. Rice GE, Munro JM, Bevilacqua MP. Inducible cell adhesion molecule 110 (INCAM-110) is an endothelial receptor for lymphocytes: a CD11/CD18-independent adhesion mechanism. *J Exp Med* 1990; **171:** 1369–1374.
108. Carlos TM, Schwartz BR, Kovach NL, *et al.* Vascular cell adhesion molecule-1 mediates lymphocyte adherence to cytokine-activated cultured human endothelial. *Blood* 1990; **76:** 965–970.
109. Osborn L, Hession C, Tizard R, *et al.* Direct expression cloning of vascular cell adhesion molecule 1, a cytokine-induced endothelial protein that binds to lymphocytes. *Cell* 1989; **59:** 1203–1211.
110. Polte T, Newman W, Gopal TV. Full length vascular cell adhesion molecule 1 (VCAM-1). *Nucleic Acids Res* 1990; **18:** 5901.
111. Hession C, Tizard R, Vassallo C, *et al.* Cloning of an alternate form of vascular cell adhesion molecule-1 (VCAM1). *J Biol Chem* 1991; **266:** 6682–6685.
112. Cybulsky MI, Fries JW, Williams AJ, *et al.* Alternative splicing of human VCAM-1 in activated vascular endothelium. *Am J Pathol* 1991; **138:** 815–820.
113. Strauch UG, Lifka A, Gosslar U, Kilshaw PJ, Clements J, Holzmann B. Distinct binding specificities of integrins alpha 4 beta 7 (LPAM-1), alpha 4 beta 1 (VLA-4), and alpha IEL beta 7. *Int Immunol* 1994; **6:** 263–275.
114. Chan BM, Elices MJ, Murphy E, Hemler ME. Adhesion to vascular cell adhesion molecule 1 and fibronectin. Comparison of alpha 4 beta 1 (VLA-4) and alpha 4 beta 7 on the human B cell line JY. *J Biol Chem* 1992; **267:** 8366–8370.
115. Elices MJ, Osborn L, Takada Y, *et al.* VCAM-1 on activated endothelium interacts with the leukocyte integrin VLA-4 at a site distinct from the VLA-4/fibronectin binding site. *Cell* 1990; **60:** 577–684.
116. Ruegg C, Postigo AA, Sikorski EE, Butcher EC, Pytela R, Erle DJ. Role of integrin alpha 4 beta 7/alpha 4 beta P in lymphocyte adherence to fibronectin and VCAM-1 and in homotypic cell clustering. *J Cell Biol* 1992; **117:** 179–189.
117. Osborn L, Vassallo C, Benjamin CD. Activated endothelium binds lymphocytes through a novel binding site in the alternately spliced domain of vascular cell adhesion molecule-1. *J Exp Med* 1992; **176:** 99–107.
118. Pepinsky B, Hession C, Chen LL, *et al.* Structure/function studies on vascular cell adhesion molecule-1. *J Biol Chem* 1992; **267:** 17820–17826.
119. Kavanaugh AF, Lightfoot E, Lipsky PE, Oppenheimer-Marks N. Role of CD11/CD18 in adhesion and transendothelial migration of T cells. Analysis utilizing CD18-deficient T cell clones. *J Immunol* 1991; **146:** 4149–4156.
120. Oppenheimer-Marks N, Davis LS, Bogue DT, Ramberg J, Lipsky PE. Differential utilization of ICAM-1 and VCAM-1 during the adhesion and transendothelial migration of human T lymphocytes. *J Immunol* 1991; **147:** 2913–2921.
121. DeLisser HM, Newman PJ, Albelda SM. Molecular and functional aspects of PECAM-1/CD31. *Immunol Today* 1994; **15:** 490–495.
122. Simmons DL, Walker C, Power C, Pigott R. Molecular cloning of CD31, a putative intercellular adhesion molecule closely related to carcinoembryonic antigen. *J Exp Med* 1990; **171:** 2147–2152.
123. Newman PJ, Berndt MC, Gorski J, *et al.* PECAM-1 (CD31) cloning and relation to adhesion molecules of the immunoglobulin gene superfamily. *Science* 1990; **247:** 1219–1222.
124. Stockinger H, Gadd SJ, Eher R, *et al.* Molecular characterization and functional analysis of the leukocyte surface protein CD31. *J Immunol* 1990; **145:** 3889–3897.
125. Albelda SM, Muller WA, Buck CA, Newman PJ. Molecular and cellular properties of PECAM-1 (endoCAM/CD31): a novel vascular cell–cell adhesion molecule. *J Cell Biol* 1991; **114:** 1059–1068.
126. Kirschbaum NE, Gumina RJ, Newman PJ. Organization of the gene for human platelet/endothelial cell adhesion molecule-1 shows alternatively spliced isoforms and a functionally complex cytoplasmic domain. *Blood* 1994; **84:** 4028–4037.
127. Muller WA, Berman ME, Newman PJ, DeLisser HM, Albelda SM. A heterophilic adhesion mechanism for platelet/endothelial cell adhesion molecule 1 (CD31). *J Exp Med* 1992; **175:** 1401–1404.
128. Newman PJ, Hillery CA, Albrecht R, *et al.* Activation-dependent changes in human platelet PECAM-1: phosphorylation, cytoskeletal association, and surface membrane redistribution. *J Cell Biol* 1992; **119:** 239–246.

129. Muller WA, Weigl SA, Deng X, Phillips DM. PECAM-1 is required for transendothelial migration of leukocytes. *J Exp Med* 1993; **178:** 449–460.
130. Franke WW, Cowin P, Grund C, Kuhn C, Kepprell HP. The endothelial junction: the plaque and its components. In: Simionescu N, Simionescu M, eds. *Endothelial Cell Biology in Health and Disease.* New York: Plenum Press, 1988: 147–166.
131. Caveda L, Corada M, Martin-Padura I, *et al.* Structural characteristics and functional role of endothelial cell to cell junctions. *Endothelium* 1994; **2:** 1–10.
132. Rubin LL. Endothelial cells: adhesion and tight junctions. *Curr Opin Cell Biol* 1992; **4:** 830–833.
133. Simionescu N, Simionescu M. Endothelial transport macromolecules: transcytosis and endocytosis. *Cell Biol Reviews* 1991; **25:** 5–80.
134. Gumbiner BM. Breaking through the tight junction barrier. *J Cell Biol* 1993; **123:** 1631–1633.
135. Anderson JM, Balda MS, Fanning AS. The structure and regulation of tight junctions. *Curr Opin Cell Biol* 1993; **5:** 772–778.
136. Risau W, Wolburg H. Development of the blood–brain barrier. *Trends Neurosci* 1990; **13:** 174–178.
137. Beyer EC. Gap junctions. *Int Rev Cytol* 1993; **137C:** 1–37.
138. Schmelz M, Franke WW. Complexus adhaerentes, a new group of desmoplakin-containing junctions in endothelial cells: the syndesmos connecting endothelial cells of lymph nodes. *Eur J Cell Biol* 1993; **61:** 274–289.
139. Huang AJ, Silverstein SC. Mechanisms of neutrophil migration across endothelium. In: Simionescu N, Simionescu M, eds. *Endothelial Cell Dysfunction.* Plenum Press: New York, 1992; 201–231.
140. Huang AJ, Manning JE, Bandak TM, Ratau MC, Hanser KR, Silverstein SC. Endothelial cell cytosolic free calcium regulates neutrophil migration across monolayers of endothelial cells. *J Cell Biol* 1993; **120:** 1371–1378.
141. Durieu-Trautmann O, Chaverot N, Cazaubon S, Strosberg AD, Couraud PO. Intercellular adhesion molecule 1 activation induces tyrosine phosphorylation of the cytoskeleton-associated protein cortactin in brain microvessel endothelial cells. *J Biol Chem* 1994; **269:** 1–4.
142. Huang AL, Jan KM, Chien S. Role of intercellular junctions in the passage of horseradish peroxidase across aortic endothelium. *Lab Invest* 1992; **67:** 201–209.

**Received 17 July 1995; accepted 1 August 1995**
